# Identification of miRNA-mRNA Crosstalk in Respiratory Syncytial Virus- (RSV-) Associated Pediatric Pneumonia through Integrated miRNAome and Transcriptome Analysis

**DOI:** 10.1155/2020/8919534

**Published:** 2020-05-01

**Authors:** Xu Zhang, Feng Huang, Diyuan Yang, Tao Peng, Gen Lu

**Affiliations:** ^1^Guangzhou Women and Children's Medical Center, Guangzhou Medical University, Guangzhou, 510120 Guangdong, China; ^2^Sino-French Hoffmann Institute, Guangzhou Medical University, Guangzhou, 510120 Guangdong, China; ^3^Institute of Human Virology, Key Laboratory of Tropical Disease Control of Ministry of Education, Guangdong Engineering Research Center for Antimicrobial Agent and Immunotechnology, Zhongshan School of Medicine, Sun Yat-sen University, Guangzhou, Guangdong, China

## Abstract

Respiratory syncytial virus (RSV) is the most common respiratory virus and is associated with pediatric pneumonia, causing bronchiolitis and significant mortality in infants and young children. MicroRNAs (miRNAs) are endogenous noncoding small RNAs that function in gene regulation and are associated with host immune response and disease progression. In the present study, we profiled the global transcriptome and miRNAome of whole blood samples from children with mild or severe RSV-associated pneumonia, aiming to identify the potential biomarkers and investigate the molecular mechanisms of severe RSV-associated pediatric pneumonia. We found that expression profiles of whole blood microRNAs and mRNAs were altered and distinctly different in children with severe RSV-associated pneumonia. In particular, the four most significantly upregulated miRNAs in children with severe RSV-associated pneumonia were hsa-miR-1271-5p, hsa-miR-10a-3p, hsa-miR-125b-5p, and hsa-miR-30b-3p. The severe RSV-associated pneumonia-specific differentially expressed miRNA target interaction network was also contrasted. These target genes were further analyzed with Gene Ontology enrichment analysis. We found that most of the target genes were involved in inflammatory and immune responses, including the NF-*κ*B signaling pathway, the MAPK signaling pathway, and T cell receptor signaling. Our findings will contribute to the identification of biomarkers and new drug design strategies to treat severe RSV-associated pediatric pneumonia.

## 1. Introduction

Pneumonia is a global respiratory disease that causes considerable morbidity and mortality in children. It has been reported that pneumonia affects nearly 1.3 million in children annually [1, 2]. Pediatric pneumonia easily recurs and usually results in severe complications due to delay or incomplete treatment [3]. Thus, it is important to study the early identification of the underlying pathogenesis and effective therapeutic targets for the treatment of pediatric pneumonia.

Respiratory syncytial virus (RSV) is the most common respiratory virus that is associated with pediatric pneumonia [4–6]. RSV, a negative-sense and single-stranded RNA virus, belongs to the *Pneumovirus* genus of the *Paramyxoviridae* family [7–9]. Clinically significantly, RSV infection occurs mainly in children and nearly all children have RSV infection between 2 and 3 years of age [10–14]. RSV infection symptoms in children range from mild upper respiratory tract infection to severe respiratory infection including bronchiolitis or pneumonia, which results in hospitalization and severe complications [11, 13, 15–17]. To date, no specific antiviral drugs or vaccine for RSV has been discovered. The traditional diagnosis of RSV pneumonia is also limited. Hence, understanding the host and virus interaction will help to improve the treatment methods and diagnosis strategies.

MicroRNAs (miRNAs) are approximately 20-25-nucleotide-long small RNAs that function in transcriptional and posttranscriptional gene regulation [18–21]. miRNAs belong to the most abundant small RNA families that are conserved across all eukaryotes and play a global regulatory role in controlling cell proliferation, cell differentiation, homeostasis, disease progression, and inflammatory responses [22, 23]. Altered expression of miRNAs has been reported to have diagnostic potential in various diseases including autoimmune diseases, cancers, and infectious diseases. Notably, integrated analysis of miRNA expression profiles and mRNA expression levels has been used to successfully identify the most prominent interactions between miRNA and mRNA [24–27]. Integrated miRNA and mRNA sequencing analysis will further highlight the important roles of miRNAs in regulating the signaling pathway network. Thus, to explore the potential driving forces and cellular pathways involved in pneumonia caused by severe RSV infection, we compared the miRNA and mRNA expression profiles in whole blood samples from children with severe RSV-associated pneumonia and children with mild RSV infection, using high-throughput sequencing. We aimed to identify candidate diagnostic biomarkers for children with severe RSV-associated pneumonia and examine the function of miRNAs in the host defense response or inflammatory response in RSV-infected children.

## 2. Materials and Methods

### 2.1. Patients

Whole blood EDTA samples were obtained from children with RSV infection that were treated at the Guangzhou Women and Children's Medical Center. The study was conducted from March 1, 2019, to September 30, 2019, and included samples from 23 children with mild RSV-associated pneumonia (denoted as Mild in the study) and 23 children with severe RSV-associated pneumonia (denoted as Severe in the study) (Table 1). RSV infection was diagnosed using real-time reverse transcription-polymerase chain reaction (RT-PCR) on nasopharyngeal swabs that were obtained at the time of admission. Patients with RSV-associated pediatric pneumonia were diagnosed clinically by symptoms and signs and confirmed radiologically and etiologically. Inclusion criteria for the participants in the study were (1) infants aged 1-12 months with (2) RSV-associated pneumonia confirmed within 7 days of symptom onset and (3) written informed consent from participants' guardians. Children were excluded in the study if (1) there was evidence of infection with other organisms in addition to RSV, (2) corticosteroids were used as part of therapy before the study, and (3) there were significant underlying comorbidities (severe malnutrition, chronic cardiac or chronic pulmonary disease). Pneumonia severity was classified according to British Thoracic Society Guidelines [28]. Severe cases were identified in the presence of at least one of the following signs: respiratory rate > 70 breaths/min, moderate to severe recessions, nasal flaring, cyanosis, grunting, inability to feed, and arterial saturations < 92%. Among the 46 patients enrolled in the study, a discovery cohort of 6 patients (3 Mild and 3 Severe) was used for high-throughput sequencing. To identify the potential diagnostic miRNA biomarkers for severe RSV-associated pneumonia, we focused on the upregulated miRNAs in children with severe RSV-associated pneumonia, and more samples (a separate verification cohort of 40 patients with 20 Mild and 20 Severe) were collected for the validation. The study was approved by the Ethics Committee at Guangzhou Women and Children's Medical Center (number 201940301), and written informed consent was obtained from all guardians.

### 2.2. RNA Extraction

RNA was extracted from whole blood samples using the TRIzol reagent (New England Biolabs) according to the manufacturer's protocol. The extracted total RNAs were used for miRNA and mRNA high-throughput sequencing (*n* = 3 Mild, *n* = 3 Severe) or quantitative real-time PCR (*n* = 20 Mild, *n* = 20 Severe).

### 2.3. High-Throughput Sequencing

miRNA sequencing was performed as previously described using the NEBNext Multiplex Small RNA Library Prep Set for Illumina Guide [29], and mRNA libraries were constructed using the NEBNext Ultra RNA Library Prep Kit for Illumina (New England Biolabs) according to the manufacturer's protocol [30]. We selected the differentially expressed mRNAs with the criterion of *P* < 0.05 and effect size > 1.5, and the differentially expressed miRNAs were selected with the criterion of *P* < 0.05 and effect size > 1.0.

### 2.4. Quantitative Real-Time PCR (qRT-PCR)

The qRT-PCR experiment was performed as previously described [29, 31]. In brief, the isolated RNAs were used to synthesize cDNA with a PrimeScript RT Reagent Kit (TaKaRa). The sequences of primers for reverse transcription are listed in Table 2. The qRT-PCR experiment was performed on a Bio-Rad CFX96 real-time PCR detection system (Bio-Rad) using SYBR Premix Ex Taq (TaKaRa) with specific primers. U6 was used as an endogenous control. The relative expression of miRNAs was normalized to U6 using the 2^-*ΔΔ*Cq^ method. The sequences of primers for qPCR are listed in Table 3.

### 2.5. Data Analysis

We used pheatmap software to perform the hierarchical clustering analysis. The parameterization used is as follows: pheatmap (union, color=colorRampPalette (rev(c(“red”,“white”,“blue”)))(100), cluster_cols=F, scale=scale_row_col, legend=T, show_rownames=showname, cellwidth=cell_widths, main=“Cluster analysis of differentially expressed sRNA”). The TPM value of the union of the differential miRNA sets of all comparison combinations in each experimental group/sample will be used for hierarchical cluster analysis. The data were row normalized.

The cluster of miRNAs was annotated using miRBase (miRBase 20, http://www.mirbase.org/). miRNA target genes were predicted using the miRanda and RNAhybrid software packages (https://bibiserv.cebitec.uni-bielefeld.de/rnahybrid/submission.html). Differentially expressed miRNA target genes in posttranscription and the target genes whose expressions were correlated with the corresponding miRNAs were selected as miRNA targets with high accuracy based on gene expression data. The regulatory networks for miRNA target genes were visualized using Cytoscape software (version 3.4.0) (http://www.cytoscape.org).

Principle component analysis (PCA) was performed with ClustVis software (https://biit.cs.ut.ee/clustvis/).

The protein-protein interaction network of the target genes of miRNAs was analyzed using the Search Tool for the Retrieval of Interacting Genes (STRING) database (http://www.string-db.org/). The interactions identified included known and predicted interactions.

To analyze the function and the potential pathway of miRNA target genes, Gene Ontology (GO) classification enrichment was performed by using the online software DAVID 6.8 (https://david.ncifcrf.gov/).

### 2.6. Statistical Analysis

Comparisons between the severe RSV and mild RSV groups were performed using the two-tailed Student's *t*-test, and statistically significant differences were classified at *P* values of <0.05 (∗), <0.01 (∗∗), and <0.001 (∗∗∗).

## 3. Results

### 3.1. Comparison of the Differentially Expressed miRNAs and mRNAs between Children with Mild or Severe RSV-Associated Pneumonia

**Figure 1:**
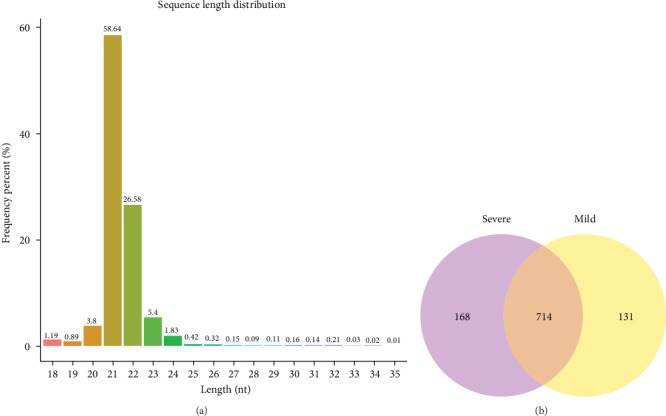
Differential expression of miRNAs in the whole blood from children with mild and severe RSV-associated pneumonia. (a) High-throughput sequencing analyzed the distribution of small RNAs in whole blood samples from children with RSV-associated pneumonia. The miRNA peaks appeared around 20–24 nt. (b) Venn diagram of differentially expressed miRNAs between severe RSV-infected and mild RSV-infected children. Mild: children with mild RSV-associated pneumonia; Severe: children with severe RSV-associated pneumonia.

**Figure 2:**
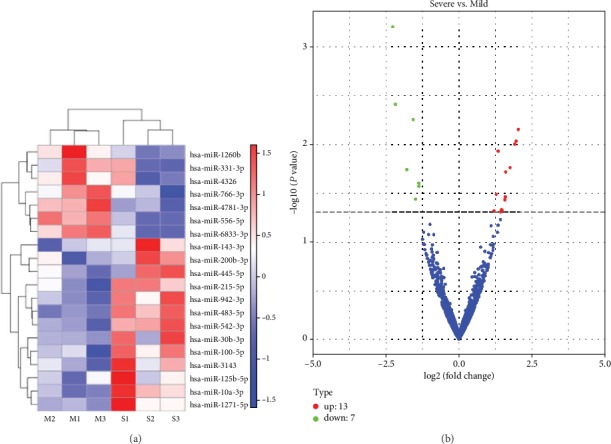
Differentially expressed miRNAs in children with severe RSV-associated pneumonia compared to children with mild RSV-associated pneumonia. (a) Hierarchical cluster analysis of differentially expressed miRNAs. (b) Volcano plot of differentially expressed miRNAs. Mild: children with mild RSV-associated pneumonia; Severe: children with severe RSV-associated pneumonia. M1, M2, and M3 represent three different children with mild RSV-associated pneumonia. S1, S2, and S3 represent three different children with severe RSV-associated pneumonia.

In the comparison of whole blood miRNA profiles between children with severe RSV- and mild RSV-associated pneumonia, we identified an apparent miRNA peak at 20-24 nt (Figure 1(a)). Furthermore, miRNAs were differentially expressed between the groups, with 168 miRNAs occurring exclusively in children with severe RSV-associated pneumonia and 131 miRNAs occurring exclusively in those with mild RSV-associated pneumonia (Figure 1(b)). In the miRNA cluster analysis, 13 miRNAs were upregulated and 7 were downregulated in all samples (Figure 2(a)) with *P* < 0.05 (Figure 2(b)).

**Figure 3:**
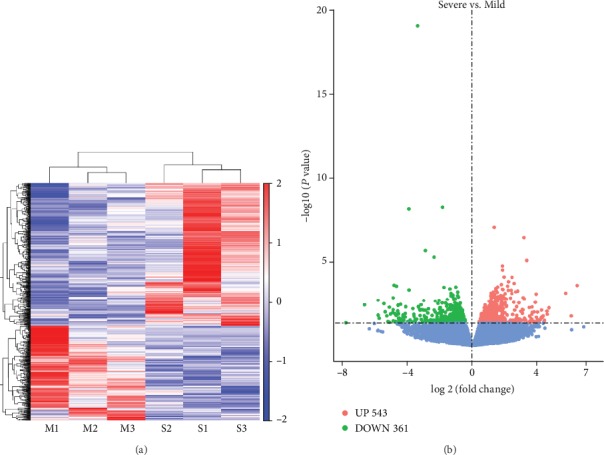
High-throughput sequencing of differentially expressed mRNAs. (a) Hierarchical cluster analysis of differentially expressed mRNAs. (b) Volcano plot of differentially expressed mRNAs. Mild: children with mild RSV-associated pneumonia; Severe: children with severe RSV-associated pneumonia.

In high-throughput sequencing analysis, we identified 543 upregulated genes and 361 that were downregulated in children with severe RSV-associated pneumonia compared to those with mild RSV-associated pneumonia (Figure 3(a)), with *P* < 0.05 (Figure 3(b)).

### 3.2. Validation of the Selected miRNAs and Identification of the Potential Diagnostic miRNA Biomarkers

**Figure 4:**
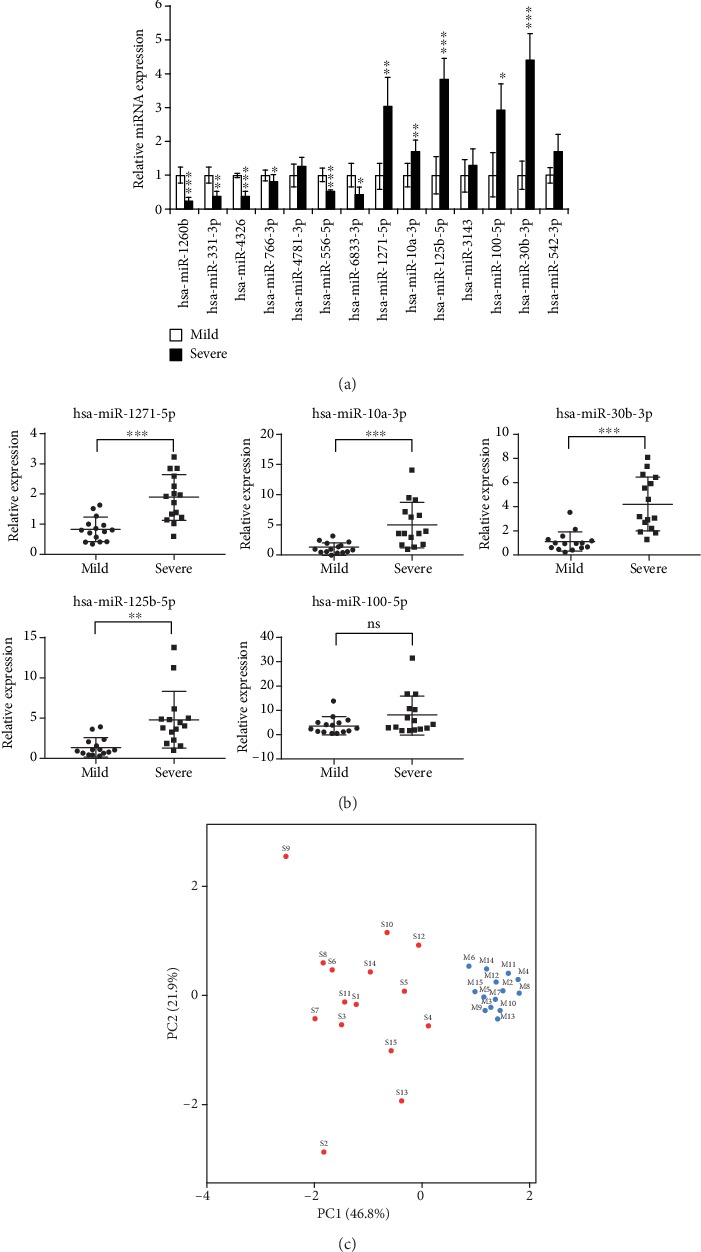
Validation of differentially expressed miRNA expression levels by qRT-PCR. (a) Relative quantities of 14 miRNAs were analyzed for differential expression between groups. Numbers in each group: mild: 5; severe: 5. (b) Quantification of the significantly upregulated miRNAs in (a). Numbers in each group: mild: 15; severe: 15. Data are shown as mean ± SD from three independent experiments. ^∗∗^*P* < 0.01 and ^∗∗∗^*P* < 0.001 (Student's *t*-test). ns: not significant. (c) The principle component analysis (PCA) of the significantly upregulated miRNAs in (b). Mild: children with mild RSV-associated pneumonia; Severe: children with severe RSV-associated pneumonia.

The differentially expressed miRNAs were further validated via qRT-PCR. Five miRNAs, namely, hsa-miR-1271-5p, hsa-miR-10a-3p, hsa-miR-125b-5p, hsa-miR-100-5p, and hsa-miR-30b-3p, were elevated in children with severe RSV-associated pneumonia. Furthermore, 6 miRNAs, including hsa-miR-1260b, hsa-miR-331-3p, hsa-miR-4326, hsa-miR-766-3p, hsa-miR-556-5p, and hsa-miR-6833-3p, were downregulated in samples from children with severe RSV-associated pneumonia compared to samples from children with mild RSV-associated pneumonia (Figure 4(a)), which were consistent with our high-throughput sequencing data. Based on the results of 15 samples from children with severe RSV-associated pneumonia and 15 samples from mild RSV-infected children, we found that hsa-miR-1271-5p, hsa-miR-10a-3p, hsa-miR-125b-5p, and hsa-miR-30b-3p were significantly increased in samples from children with severe RSV-associated pneumonia (Figure 4(b)). Notably, when we performed the principle component analysis (PCA) based on the expression of hsa-miR-1271-5p, hsa-miR-10a-3p, hsa-miR-125b-5p, and hsa-miR-30b-3p in the qRT-PCR result in Figure 4(b), we found that the samples from children with severe RSV-associated pneumonia could be separated from samples from children with mild RSV-associated pneumonia (Figure 4(c)). Taken together, these results indicate that hsa-miR-1271-5p, hsa-miR-10a-3p, hsa-miR-125b-5p, and hsa-miR-30b-3p may reflect severe RSV-associated pneumonia, and they may be the potential candidate biomarkers for severe RSV-associated pneumonia.

### 3.3. Prediction and Identification of Target Genes of the Differentially Expressed miRNAs

**Figure 5:**
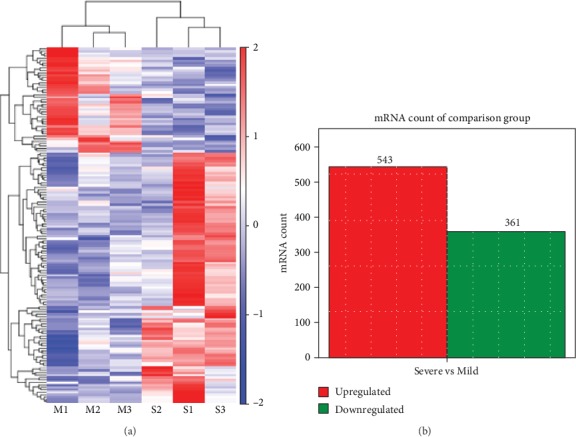
Overlapping genes from the predicted miRNA targets and differentially expressed mRNAs. (a) Hierarchical cluster analysis of overlapping genes. (b) mRNA counts of upregulated and downregulated overlapping genes. Mild: children with mild RSV-associated pneumonia; Severe: children with severe RSV-associated pneumonia. M1, M2, M3, S1, S2, and S3 are the same patients as in Figure 3.

**Figure 6:**
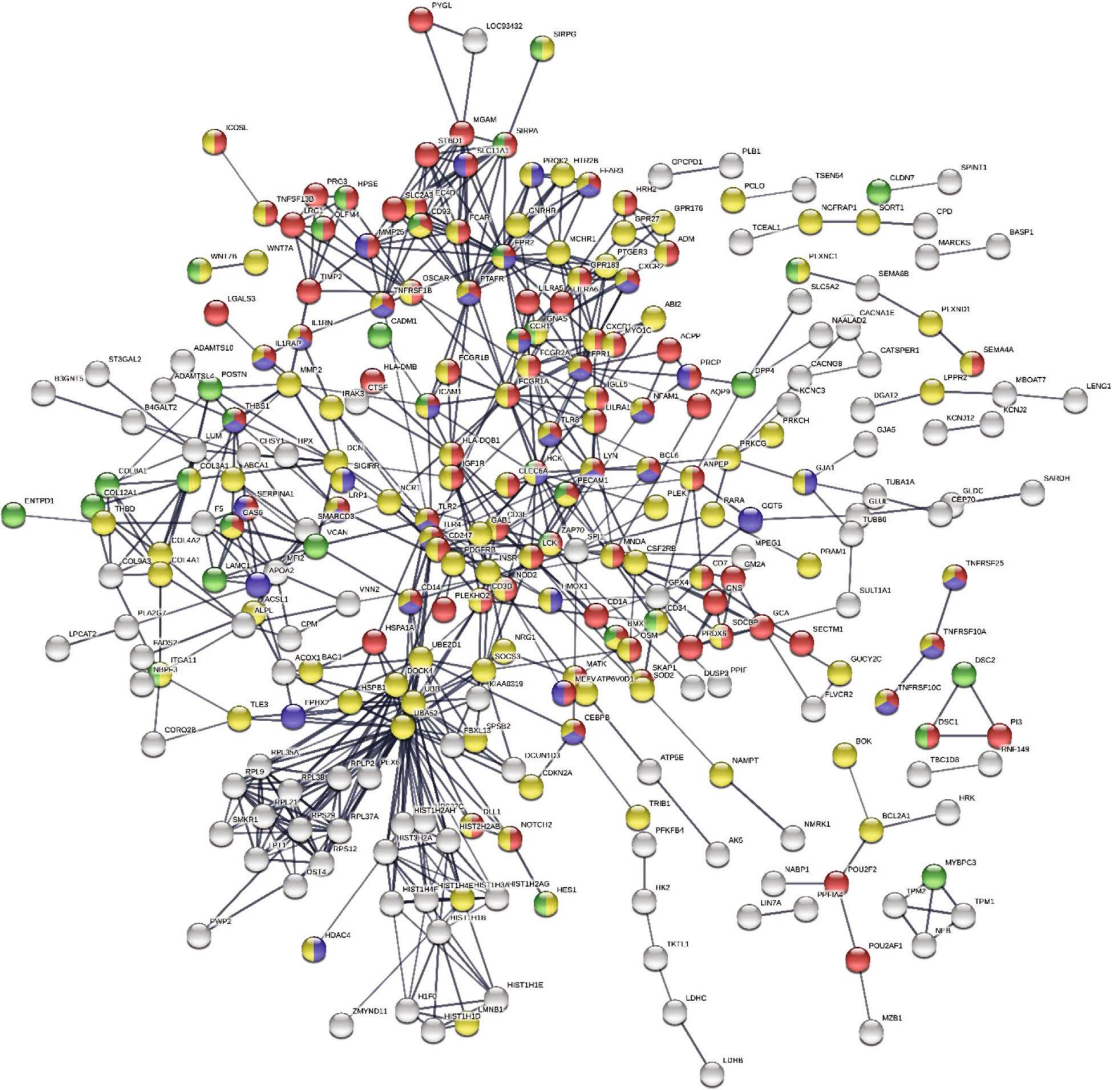
The protein-protein interaction network of target genes. The protein-protein interaction network was drawn using the STRING online tool. The minimum required interaction score was 0.7 (high confidence). Red nodes represent immune response. Blue nodes represent inflammatory response. Yellow nodes represent signal transduction. Green nodes represent cell adhesion. White nodes represent genes from the other pathways.

**Figure 7:**
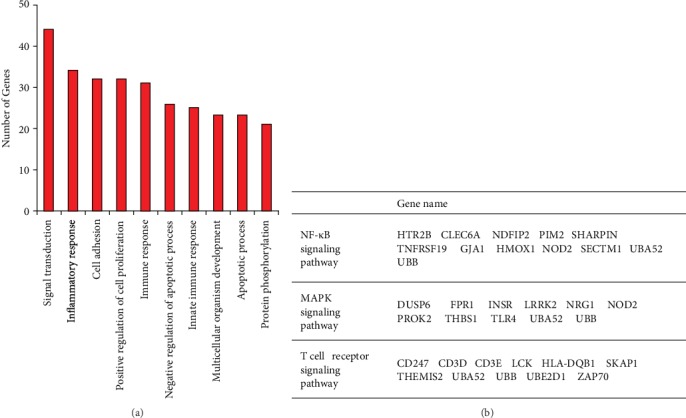
GO enrichment of target genes for miRNAs. (a) The GO enrichment of target genes for miRNAs between groups. (b) Target genes from the NF-*κ*B signaling pathway, the MAPK signaling pathway, and the T cell receptor signaling pathway.

**Figure 8:**
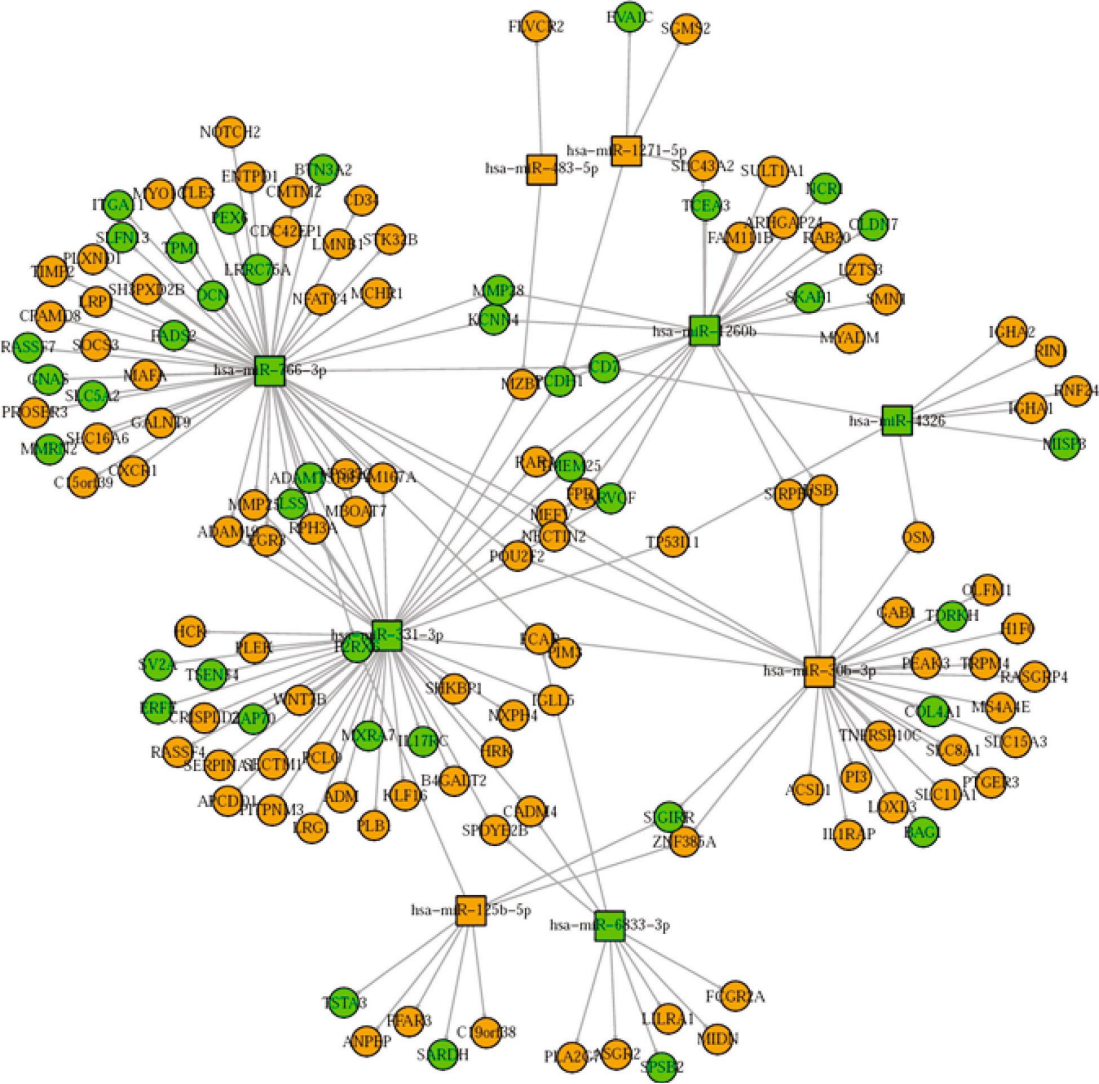
The regulatory miRNA target gene network. The regulatory miRNA gene network was analyzed using Cytoscape software. Squares and circles represent miRNA and target genes, respectively. Orange nodes represent genes that are upregulated in severe RSV-associated pneumonia, and blue nodes represent downregulated genes.

To further examine the possible molecular mechanisms of the differentially expressed miRNAs in severe RSV-associated pediatric pneumonia, we performed RNA sequencing and integrated miRNA profile and transcriptome analysis. We combined the differentially expressed mRNAs and miRNAs with miRNA target predictions to obtain genuine miRNA targets. As a result, 543 upregulated plus 361 downregulated genes formed the miRNA target gene pairs with an inverse correlation of expression (Figure 5(a)). Furthermore, 543 miRNA target gene pairs were identified for the upregulated miRNA, while 361 miRNA target gene pairs were identified for the downregulated miRNA (Figure 5(b)). We constructed a protein-protein interaction network of these overlapping genes, in which the nodes represent the proteins and the edges depict their associations (Figure 6). When we selected these genes for GO annotations, we found that most of these genes were involved in signal transduction and the inflammatory and immune responses (including innate immune response) (Figures 6 and 7(a)), indicating that severe RSV infection caused active inflammatory and immune responses that resulted in pneumonia in children. Notably, most of these genes were related to the NF-*κ*B signaling pathway, the MAPK signaling pathway, and the T cell receptor signaling pathway (Figure 7(b)), including TNFRSF19, HMOX1, TLR4, LCK, and ZAP70. We further established the miRNA gene regulatory networks from the above miRNA gene pairs using Cytoscape software (Figure 8). These genes may reflect the mechanism of severe RSV-associated pneumonia, in which the NF-*κ*B and MAPK signaling pathways play important roles.

## 4. Discussion

Human RSV is a common cause of hospitalization and acute respiratory infections in children [12, 32]. RSV-associated respiratory infection is usually thought to be mild and self-limited. However, RSV infection can cause severe pneumonia with high fatality rates and permanent lung damage in some patients. RSV can persistently infect humans, especially children [3, 33]. The mainstay of treatment for RSV-associated pneumonia is still limited based on no apparently effective approved antiviral drugs. There is still a lack of accurate assessment tools and biomarkers for severe RSV-associated pneumonia. In present, the common methods of assessing the severity of RSV pneumonia are based on clinical characteristics. However, these methods are overdue and are unable to provide an effective identification of severe RSV-associated pneumonia. Moreover, the underlying mechanisms of the pathogenesis of severe RSV-associated pneumonia in children remain incompletely understood. Thus, it is important to investigate the mechanism of severe RSV-associated pneumonia and identify the biomarkers for severe RSV-associated pediatric pneumonia. In the present study, we explored the potential roles of miRNAs in children with severe RSV-associated pneumonia and demonstrated significantly different miRNA and transcriptome responses, compared to children with mild RSV-associated pneumonia. miRNA and transcriptome responses in RSV-associated pneumonia may reflect the associated pathology and provide a better understanding of the disease.

miRNA is a global regulatory network, and it has been reported to control homeostasis, cell proliferation, cell differentiation, disease progression, and inflammatory responses. Altered miRNA profiles and their diagnostic potential are associated with various disorders, autoimmune diseases, and infectious diseases [34, 35]. miRNA profiles from RSV-infected biofluids, whole blood, and tissue samples have been assessed in previous studies [36–40]. However, previous studies were aimed at discovering biomarkers for RSV infection, and none of them focused on miRNA profiles in patients with severe RSV-associated pneumonia. In our study, by comparing with samples from mild RSV-infected children, we were able to analyze the miRNA expression profile of severe RSV-associated pediatric pneumonia. Furthermore, to our knowledge, this is the first study to integrate miRNA profiling and transcriptome sequencing of RSV-associated pediatric pneumonia. Combining miRNA and transcriptome sequencing will allow us to better understand the potential driving forces and cellular pathways involved in severe RSV-associated pediatric pneumonia.

Using high-throughput sequencing, we found that 168 and 131 miRNAs were differentially expressed in blood samples of severe RSV-associated pediatric pneumonia vs. blood samples of mild RSV-associated pediatric pneumonia. Among them, there were 13 upregulated miRNAs and 7 downregulated miRNAs in the 6 samples that passed the fold change filter. Those with the greatest differences and upregulation in samples from children with severe RSV-associated pneumonia were chosen for further verification. In particular, the expression levels of hsa-miR-1271-5p, hsa-miR-10a-3p, hsa-miR-125b-5p, and hsa-miR-30b-3p in 15 samples of severe RSV-associated pediatric pneumonia were significantly higher than the corresponding expression levels in 15 mild RSV-infected controls, indicating that these miRNAs could be considered good diagnostic biomarkers for severe RSV-associated pneumonia. However, based on the limited number and the regional source of the enrolled samples in the study, more experiments with additional samples should be performed to confirm the diagnostic capabilities of the miRNAs identified in the study.

To further explore the possible molecular mechanisms of the differentially expressed miRNAs in severe RSV-associated pediatric pneumonia, we performed RNA sequencing and integrated miRNA profile and transcriptome analysis. Through GO enrichment analysis of the target genes of miRNAs, we showed that most target genes were involved in the NF-*κ*B and MAPK signaling pathways. Notably, NF-*κ*B and MAPK signaling pathways are crucial components of many immune responses in humans [41, 42]. Inflammatory responses involve various receptors of the MAPK signaling pathway to integrate a danger and/or injury signal to transduce NF-*κ*B activation [43]. The secretion of many proinflammatory cytokines by macrophages or dendritic cells (DCs) relies on the NF-*κ*B signaling pathway, indicating that activation of NF-*κ*B signaling will result in increased production of inflammatory cytokines, which may lead to pneumonia [41, 44–47]. Also, NF-*κ*B is an important antiapoptotic transcription factor for immune cells such as neutrophils, which plays an important role in wound repair during infection and inflammation [45]. Thus, activation of NF-*κ*B signaling may result in severe complications during severe RSV infection. However, the underlying mechanisms of the critical target genes identified in the study remain to be clarified. The NF-*κ*B signaling pathway has received attention for the development of therapies, which will create a new strategy for the treatment of severe RSV-associated pediatric pneumonia.

## Figures and Tables

**Table 1:** Characteristics of patients with mild and severe RSV-associated pediatric pneumonia.

Clinical information	Mild (*n* = 23)	Severe (*n* = 23)	*P* value
Male gender, *n* (%)	14 (60.9%)	13 (56.5%)	0.765
Age (months)	6.22 ± 3.63	5.96 ± 3.78	0.813
Underlying comorbidities, *n* (%)	2 (8.7%)	8 (34.8%)	0.074
Clinical presentation, *n* (%)			
Fever	23 (100%)	23 (100%)	1.000
Physical examination, *n* (%)			
Tachypnea^a^	0 (0%)	20 (87.0%)	0.000
Laboratory examination^b^			
White blood cell (×10^9^/L)^c^	9.58 ± 3.25	9.43 ± 4.08	0.893
C-reactive protein (CRP)^d^ (mg/L)	9.00 (5.00-19.79)	14.20 (11.00-19.00)	0.030
Radiology, *n* (%)			
Patchypacity^e^	23 (100%)	23 (100%)	1.000
Management			
Oxygen therapy, *n* (%)	0 (0%)	20 (87.0%)	0.000
Ventilator support, *n* (%)	0 (0%)	3 (13.0%)	0.233
Duration of wheezing^f^ (days)	5.96 ± 1.89	9.48 ± 3.54	0.000
Duration of hospital stay (days)	7.00 (5.00-8.00)	11.00 (8.00-16.00)	0.000

Data was presented as number (percentage), median (25th-75th percentile), or mean ± standard deviation, where appropriate, and compared with the chi-squared test, independent-samples *t*-test, and Mann-Whitney *U* test, respectively. For all analyses, 2-tailed *P* values were calculated by IBM SPSS statistics 25.0. Statistical significance was defined as *P* < 0.05. ^a^Respiratory rate > 70 breaths/min. ^b^Data extracted from the first test for the children on admission. ^c^The normal reference value was (5–12) × 10^9^/L. ^d^The normal reference value was (0-3) mg/L. ^e^Judged by chest radiograph or CT scan in the whole course of the patients. ^f^Wheezing duration from onset to relief.

**Table 2:** Specific reverse transcription primer sequences used for miRNAs.

miRNA	Reverse transcription primer sequence (5′-3′)
hsa-miR-1260b	GTCGTATCCAGTGCAGGGTCCGAGGTATTCGCACTGGATACGACGGTG
hsa-miR-331-3p	GTCGTATCCAGTGCAGGGTCCGAGGTATTCGCACTGGATACGACTCTAG
hsa-miR-4326	GTCGTATCCAGTGCAGGGTCCGAGGTATTCGCACTGGATACGACGTCTGG
hsa-miR-766-3p	GTCGTATCCAGTGCAGGGTCCGAGGTATTCGCACTGGATACGACGCTGAG
hsa-miR-4781-3p	GTCGTATCCAGTGCAGGGTCCGAGGTATTCGCACTGGATACGACCTCTAG
hsa-miR-556-5p	GTCGTATCCAGTGCAGGGTCCGAGGTATTCGCACTGGATACGACCTCATA
hsa-miR-6833-3p	GTCGTATCCAGTGCAGGGTCCGAGGTATTCGCACTGGATACGACCTGAGG
hsa-miR-1271-5p	GTCGTATCCAGTGCAGGGTCCGAGGTATTCGCACTGGATACGACTGAGTG
hsa-miR-10a-3p	GTCGTATCCAGTGCAGGGTCCGAGGTATTCGCACTGGATACGACTATTCC
hsa-miR-125b-5p	GTCGTATCCAGTGCAGGGTCCGAGGTATTCGCACTGGATACGACTCACAA
hsa-miR-3143	GTCGTATCCAGTGCAGGGTCCGAGGTATTCGCACTGGATACGACCGAAAG
hsa-miR-100-5p	GTCGTATCCAGTGCAGGGTCCGAGGTATTCGCACTGGATACGACCACAAG
hsa-miR-30b-3p	GTCGTATCCAGTGCAGGGTCCGAGGTATTCGCACTGGATACGACGAAGTA
hsa-miR-542-3p	GTCGTATCCAGTGCAGGGTCCGAGGTATTCGCACTGGATACGACTTTCAG

**Table 3:** Primers for qPCR.

miRNA or U6	Primer name	Sequence
hsa-miR-1260b	hsa-miR-1260b-F	ATCCCACCACTGCCACCAT
	hsa-miR-1260b-R	GTGCAGGGTCCGAGGT

hsa-miR-331-3p	hsa-miR-331-3p-F	GCCCCTGGGCCTATCCTAGAA
	hsa-miR-331-3p-R	GTGCAGGGTCCGAGGT

hsa-miR-4326	hsa-miR-4326-F	TGTTCCTCTGTCTCCCAGAC
	hsa-miR-4326-R	GTGCAGGGTCCGAGGT

hsa-miR-766-3p	hsa-miR-766-3p-F	ACTCCAGCCCCACAGCCTCAGC
	hsa-miR-766-3p-R	GTGCAGGGTCCGAGGT

hsa-miR-4781-3p	hsa-miR-4781-3p-F	AATGTTGGAATCCTCGCTAGAG
	hsa-miR-4781-3p-R	GTGCAGGGTCCGAGGT

hsa-miR-556-5p	hsa-miR-556-5p-F	GATGAGCTCATTGTAATATGAG
	hsa-miR-556-5p-R	GTGCAGGGTCCGAGGT

hsa-miR-6833-3p	hsa-miR-6833-3p-F	TTTCTCTCTCCACTTCCTCAG
	hsa-miR-6833-3p-R	GTGCAGGGTCCGAGGT

hsa-miR-1271-5p	hsa-miR-1271-5p-F	CTTGGCACCTAGCAAGCACTCA
	hsa-miR-1271-5p-R	GTGCAGGGTCCGAGGT

hsa-miR-10a-3p	hsa-miR-10a-3p-F	CAAATTCGTATCTAGGGGAATA
	hsa-miR-10a-3p-R	GTGCAGGGTCCGAGGT

hsa-miR-125b-5p	hsa-miR-125b-5p-F	TCCCTGAGACCCTAACTTGTGA
	hsa-miR-125b-5p-R	GTGCAGGGTCCGAGGT

hsa-miR-3143	hsa-miR-3143-F	ATAACATTGTAAAGCGCTTCTTTCG
	hsa-miR-3143-R	GTGCAGGGTCCGAGGT

hsa-miR-100-5p	hsa-miR-100-5p-F	AACCCGTAGATCCGAACTTGTG
	hsa-miR-100-5p-R	GTGCAGGGTCCGAGGT

hsa-miR-30b-3p	hsa-miR-30b-3p-F	CTGGGAGGTGGATGTTTACTTC
	hsa-miR-30b-3p-R	GTGCAGGGTCCGAGGT

hsa-miR-542-3p	hsa-miR-542-3p-F	TGTGACAGATTGATAACTGAAA
	hsa-miR-542-3p-R	GTGCAGGGTCCGAGGT

U6	U6-F	CAGCACATATACTAAAATTGGAACG
	U6-R	ACGAATTTGCGTGTCATCC

## Data Availability

The data used to support the findings of this study are included within the article.

## References

[1] Agweyu A., Kibore M., Digolo L. (2014). Prevalence and correlates of treatment failure among Kenyan children hospitalised with severe community-acquired pneumonia: a prospective study of the clinical effectiveness of WHO pneumonia case management guidelines. *Tropical Medicine & International Health*.

[2] Bhutta Z. A., Das J. K., Walker N. (2013). Interventions to address deaths from childhood pneumonia and diarrhoea equitably: what works and at what cost?. *The Lancet*.

[3] Jadavji T., Law B., Lebel M. H., Kennedy W. A., Gold R., Wang E. E. (1997). A practical guide for the diagnosis and treatment of pediatric pneumonia. *CMAJ: Canadian Medical Association Journal = Journal de l'Association Medicale Canadienne*.

[4] Higdon M. M., le T., O'Brien K. L. (2017). Association of C-reactive protein with bacterial and respiratory syncytial virus-associated pneumonia among children aged <5 years in the PERCH study. *Clinical Infectious Diseases: An Official Publication of the Infectious Diseases Society of America*.

[5] Cooper A. C., Banasiak N. C., Allen P. J. (2003). Management and prevention strategies for respiratory syncytial virus (RSV) bronchiolitis in infants and young children: a review of evidence-based practice interventions. *Pediatric Nursing*.

[6] Nair H., Verma V. R., Theodoratou E. (2011). An evaluation of the emerging interventions against respiratory syncytial virus (RSV)-associated acute lower respiratory infections in children. *BMC Public Health*.

[7] Al-Sonboli N., Hart C. A., Al-Aghbari N., Al-Ansi A., Ashoor O., Cuevas L. E. (2006). Human metapneumovirus and respiratory syncytial virus disease in children, Yemen. *Emerging Infectious Diseases*.

[8] Hermos C. R., Vargas S. O., McAdam A. J. (2010). Human metapneumovirus. *Clinics in Laboratory Medicine*.

[9] Papenburg J., Boivin G. (2010). The distinguishing features of human metapneumovirus and respiratory syncytial virus. *Reviews in Medical Virology*.

[10] Glezen W. P., Taber L. H., Frank A. L., Kasel J. A. (1986). Risk of primary infection and reinfection with respiratory syncytial virus. *American Journal of Diseases of Children*.

[11] Leader S., Kohlhase K. (2003). Recent trends in severe respiratory syncytial virus (RSV) among US infants, 1997 to 2000. *The Journal of Pediatrics*.

[12] Welliver R. C. (2003). Review of epidemiology and clinical risk factors for severe respiratory syncytial virus (RSV) infection. *The Journal of Pediatrics*.

[13] Kneyber M. C. J., Steyerberg E. W., de Groot R., Moll H. A. (2000). Long-term effects of respiratory syncytial virus (RSV) bronchiolitis in infants and young children: a quantitative review. *Acta Paediatrica*.

[14] Leung A. K. C., Wong A. H. C., Hon K. L. (2018). Community-acquired pneumonia in children. *Recent Patents on Inflammation & Allergy Drug Discovery*.

[15] Chan M., Park J. J., Shi T. (2017). The burden of respiratory syncytial virus (RSV) associated acute lower respiratory infections in children with Down syndrome: a systematic review and meta-analysis. *Journal of Global Health*.

[16] Scheltema N. M., Gentile A., Lucion F. (2017). Global respiratory syncytial virus-associated mortality in young children (RSV GOLD): a retrospective case series. *The Lancet Global Health*.

[17] Stein R. T., Bont L. J., Zar H. (2017). Respiratory syncytial virus hospitalization and mortality: systematic review and meta-analysis. *Pediatric Pulmonology*.

[18] Llave C., Xie Z., Kasschau K. D., Carrington J. C. (2002). Cleavage of scarecrow-like mRNA targets directed by a class of Arabidopsis miRNA. *Science*.

[19] Iwakawa H. O., Tomari Y. (2015). The functions of microRNAs: mRNA decay and translational repression. *Trends in Cell Biology*.

[20] Bagga S., Bracht J., Hunter S. (2005). Regulation by let-7 and lin-4 miRNAs results in target mRNA degradation. *Cell*.

[21] Fabian M. R., Cieplak M. K., Frank F. (2011). miRNA-mediated deadenylation is orchestrated by GW182 through two conserved motifs that interact with CCR4-NOT. *Nature Structural & Molecular Biology*.

[22] Bartel D. P. (2004). MicroRNAs: genomics, biogenesis, mechanism, and function. *Cell*.

[23] Bushati N., Cohen S. M. (2007). microRNA functions. *Annual Review of Cell and Developmental Biology*.

[24] Li Z., Tzeng C.-M., Lamandé S. R. (2018). Integrated analysis of miRNA and mRNA expression profiles to identify miRNA targets. *mRNA Decay: Methods and Protocols*.

[25] Xu P., Wang J., Sun B., Xiao Z. (2018). Integrated analysis of miRNA and mRNA expression data identifies multiple miRNAs regulatory networks for the tumorigenesis of colorectal cancer. *Gene*.

[26] Zhao C., Zhang G., Yin S. (2017). Integrated analysis of mRNA-seq and miRNA-seq reveals the potential roles of sex-biased miRNA-mRNA pairs in gonad tissue of dark sleeper (*Odontobutis potamophila*). *BMC Genomics*.

[27] Diaz G., Zamboni F., Tice A., Farci P. (2015). Integrated ordination of miRNA and mRNA expression profiles. *BMC Genomics*.

[28] Principi N., Esposito S. (2011). Management of severe community-acquired pneumonia of children in developing and developed countries. *Thorax*.

[29] Huang F., Zhang J., Yang D. (2018). MicroRNA expression profile of whole blood is altered in adenovirus-infected pneumonia children. *Mediators of Inflammation*.

[30] Jia H. L., Zeng X. Q., Huang F. (2018). Integrated microRNA and mRNA sequencing analysis of age-related changes to mouse thymic epithelial cells. *IUBMB Life*.

[31] Huang F., Bai J., Zhang J. (2019). Identification of potential diagnostic biomarkers for pneumonia caused by adenovirus infection in children by screening serum exosomal microRNAs. *Molecular Medicine Reports*.

[32] Gupta S., Shamsundar R., Shet A., Srinivasa H. (2013). Rapid detection of respiratory syncytial virus (RSV) in children with acute lower respiratory tract infections: a pilot evaluation of an immuno-chromatographic rapid antigen detection method. *Clinical Laboratory*.

[33] Yeshuroon-Koffler K., Shemer-Avni Y., Keren-Naus A., Goldbart A. D. (2015). Detection of common respiratory viruses in tonsillar tissue of children with obstructive sleep apnea. *Pediatric Pulmonology*.

[34] Hebert S. S., De Strooper B. (2009). Alterations of the microRNA network cause neurodegenerative disease. *Trends in Neurosciences*.

[35] Persengiev S. P. (2012). miRNAs at the crossroad between hematopoietic malignancies and autoimmune pathogenesis. *Discovery Medicine*.

[36] Inchley C. S., Sonerud T., Fjaerli H. O., Nakstad B. (2015). Nasal mucosal microRNA expression in children with respiratory syncytial virus infection. *BMC Infectious Diseases*.

[37] Rossi G. A., Silvestri M., Colin A. A. (2015). Respiratory syncytial virus infection of airway cells: role of microRNAs. *Pediatric Pulmonology*.

[38] Banos-Lara M. D. R., Zabaleta J., Garai J., Baddoo M., Guerrero-Plata A. (2018). Comparative analysis of miRNA profile in human dendritic cells infected with respiratory syncytial virus and human metapneumovirus. *BMC Research Notes*.

[39] Wang S., Liu P., Yang P., Zheng J., Zhao D. (2017). Peripheral blood microRNAs expression is associated with infant respiratory syncytial virus infection. *Oncotarget*.

[40] Hasegawa K., Pérez-Losada M., Hoptay C. E. (2018). RSV vs. rhinovirus bronchiolitis: difference in nasal airway microRNA profiles and NF*κ*B signaling. *Pediatric Research*.

[41] Lawrence T. (2009). The nuclear factor NF-kappaB pathway in inflammation. *Cold Spring Harbor Perspectives in Biology*.

[42] Arthur J. S. C., Ley S. C. (2013). Mitogen-activated protein kinases in innate immunity. *Nature Reviews Immunology*.

[43] Cuadrado A., Nebreda A. R. (2010). Mechanisms and functions of p38 MAPK signalling. *The Biochemical Journal*.

[44] Vallabhapurapu S., Karin M. (2009). Regulation and function of NF-*κ*B transcription factors in the immune system. *Annual Review of Immunology*.

[45] Li Q., Verma I. M. (2002). NF-kappaB regulation in the immune system. *Nature Reviews Immunology*.

[46] Dong L., Zhou Y., Zhu Z. Q. (2017). Soluble epoxide hydrolase inhibitor suppresses the expression of triggering receptor expressed on myeloid cells-1 by inhibiting NF-kB activation in murine macrophage. *Inflammation*.

[47] Coleman F. T., Blahna M. T., Kamata H. (2017). Capacity of pneumococci to activate macrophage nuclear factor *κ*B: influence on necroptosis and pneumonia severity. *The Journal of Infectious Diseases*.

